# Aluminum/Carbon Composites Materials Fabricated by the Powder Metallurgy Process

**DOI:** 10.3390/ma12244030

**Published:** 2019-12-04

**Authors:** Amélie Veillère, Hiroki Kurita, Akira Kawasaki, Yongfeng Lu, Jean-Marc Heintz, Jean-François Silvain

**Affiliations:** 1 University of Bordeaux, CNRS, Bordeaux INP, ICMCB, UPR9048, F-33600 Pessac, France; 2 Department of Materials Processing, Graduate School of Engineering, Tohoku University, Sendai 980-8579, Japan; 3 Department of Electrical and Computer Engineering, University of Nebraska-Lincoln, Lincoln, NE 68588, USA

**Keywords:** Al/C composite materials, carbon fiber, diamond particle, semi-liquid route, thermal management, powder processing

## Abstract

Aluminum matrix composites reinforced with carbon fibers or diamond particles have been fabricated by a powder metallurgy process and characterized for thermal management applications. Al/C composite is a nonreactive system (absence of chemical reaction between the metallic matrix and the ceramic reinforcement) due to the presence of an alumina layer on the surface of the aluminum powder particles. In order to achieve fully dense materials and to enhance the thermo-mechanical properties of the Al/C composite materials, a semi-liquid method has been carried out with the addition of a small amount of Al-Si alloys in the Al matrix. Thermal conductivity and coefficient of thermal expansion were enhanced as compared with Al/C composites without Al-Si alloys and the experimental values were close to the ones predicted by analytical models.

## 1. Introduction

In the field of power electronics and transportation (automotive, aeronautic, and aerospace) industries, the continuous progress on the electronics components in terms of power, frequency, and miniaturization leads to more heat generation per device. Therefore, improvement of the thermal management is required to increase the performance and reliability of this kind of devices. The thermal management is realized by a heat sink material which have to present a high thermal conductivity (TC) and a tailored coefficient of thermal expansion (CTE) to reduce the thermal stresses between the different layers of the device (semiconductor, ceramic substrate, and heat sink material) [1,2,3].

For the last 40 years, a lot of studies have been achieved on metal matrix composite (MMC), such as Copper/Carbon and Aluminum/Carbon systems, in order to improve the thermal and thermo-mechanical properties of heat sink materials [4,5,6]. Micrometric carbon reinforcements such as graphite flakes and particles, diamond particles (DP) and carbon fibers (CF) have proved to be promising thermal management reinforcements due to their high TC and low CTE properties. Nanometric carbon reinforcements such as carbon nanotubes, graphene, or nano-diamonds have also been used to fabricate MMC heat sinks due to their outstanding thermal properties. Nevertheless, the improvement of the thermal properties of these composites is limited by many technical problems such as the dispersion of the nanometric reinforcements in the matrix or the high interfacial matrix-reinforcement thermal resistance [7,8].

For a lightweight heat sink, especially for embedded devices, Al is superior than copper as a matrix, not only because of its low density, but also due to its low price and low melting point.

The transfer of properties between the matrix and the reinforcement, in MMC, is correlated with the properties of the interfacial zone. When chemical bondings are obtained between the two materials, a good property transfer is expected [4,9]. Unfortunately, the Al/C system is a non-reactive system due to the natural presence of alumina (Al_2_O_3_) layer on the surface of the Al particles. This layer disturbs the sintering process and avoids the formation of aluminum carbide (Al_4_C_3_) at the Al–CF interface and therefore limits the densification behavior of Al/C composites, which is critical for their final thermal properties.

To overcome the presence of this alumina layer, many liquid-phase methods (e.g., infiltration, stir casting) have been developed. These methods improve the wettability between matrix and reinforcement and allow the fabrication of MMC with high reinforcement volume ratio and low porosity ratio. However, during the fabrication process, a large amount of Al_4_C_3_ phase is formed at the matrix–reinforcement interface. Due to its low thermal conductivity and intrinsic brittleness, the formation of excessive Al_4_C_3_ interfacial phase is detrimental to the final properties and reliability of the Al/C composite materials [10].

Therefore, the fabrication process of this composite should be well-controlled in order to obtain the required properties. In this work, we focused our investigations on the fabrication of composite materials using a semi-liquid process (liquid phase sintering). An aluminum–silicon alloy is used as the liquid phase during the sintering process. First, the melting temperature of Al-Si is lower than those of pure Al, which is needed for a semi-liquid process. Second, the use of Si addition on Al/C composite materials has been shown to have a positive effect on thermal conductivity and thermal expansion [10].

The results presented and discussed hereafter are related to the density, microstructure, and thermophysical properties of Al/C composites with two different carbon reinforcements, i.e., carbon fibers and diamond particles.

## 2. Materials and Methods

### 2.1. Composite Materials

**Figure 1:**
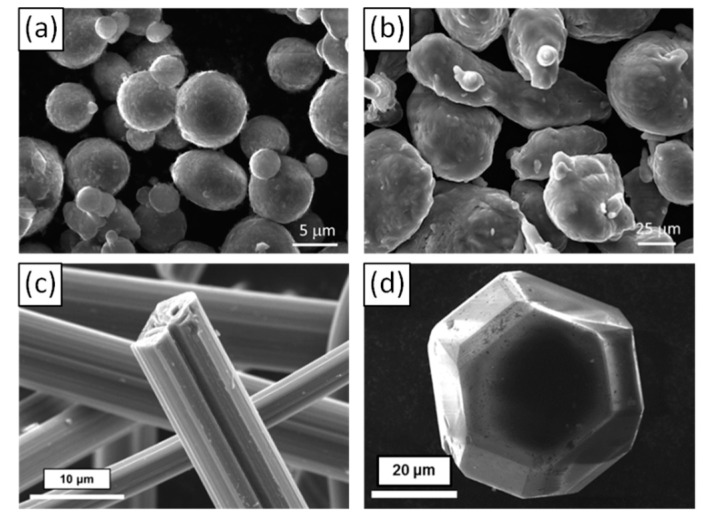
SEM micrographs of (**a**) Al powder, (**b**) Al-Si powder, (**c**) CF and (**d**) DP.

The investigated composite materials are composed of an Al-based matrix and carbon reinforcements. The Al-based matrix is composed of spherical Al powder, prepared by atomization process (F3731, Hermillon Powders, Hermillon, France) with an average diameter of 8 µm (Figure 1a) and Al-Si alloys powder with a composition of 11.3 at.% of silicon (F2071, Hermillon Powders) (Figure 1b). The melting point of Al and Al-Si is respectively 660 °C and 584.6 °C.

Two kinds of carbon reinforcements have been selected in this work:

- Pitch-based carbon fibers (Raheama, R-A301, Teijin Limited, Chiyoda, Tokyo, Japan) with an average length of 200 µm, a diameter of 10 µm and a TC of 600 W·m^−1^·K^−1^ in the longitudinal fiber direction (Figure 1c).
- Single crystal diamond powders (MBD6 quality grade from Henan Zhongxin Industry, Henan, China) with hexagonal or cubo-octahedral shapes and an average diameter of 65 μm (Figure 1d).

### 2.2. Fabrication Process

Matrix (Al and/or Al-Si) and carbon reinforcements (CF or DP) powders are mechanically mixed for 5 min in air. Two sets of composite materials were considered:

- Set A: Al/C (CF or DP) composites fabricated without Al-Si powder and used as a reference.
- Set B: (Al + Al-Si)/C (CF or DP) composites fabricated with the addition of 5 vol.% of Al-Si powder.

The fraction of reinforcement was fixed to 10, 20, 30, 40, and 50 vol.%. The mixed composite powders were then hot-pressed at 600 °C or 640 °C under 60 MPa for 30 min. The chamber was under vacuum to prevent oxidation during both heating and cooling. A temperature of 600 °C was always used for CF reinforcements while two sintering temperature (600 °C or 640 °C) were tested for DP reinforcements.

**Figure 2:**
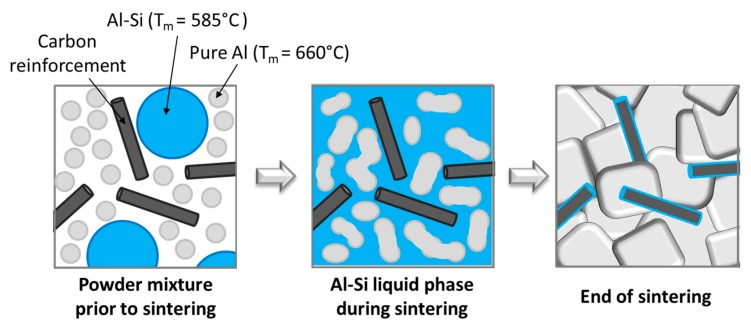
Schematic of the semi-liquid process.

Due to the chosen sintering temperatures, set A samples were fabricated by a regular powder metallurgy process while set B samples were obtained using a semi-liquid process. Indeed, the sintering temperature was chosen between the melting temperatures of pure Al and Al–Si alloy. A schematic of the semi-liquid process is shown in Figure 2.

### 2.3. Density, Microstructural, and Chemical Characterizations

The theoretical density of the composites was calculated using a rule of mixture:

(1) $$\rho_{C}=\;\rho_{m}V_{m}+\rho_{r}V_{r},$$

where *ρ_m_*, *ρ_r_* and *V_m_*, *V_r_* are the densities and the volume fractions of the matrix and the reinforcement, respectively. Experimental density was carried out using Archimedes’ method and the relative density was then calculated as the ratio of experimental and theoretical densities.

Microstructural characterization of the Al/CF composite was carried out through scanning electron microscopy (SEM; Tescan, VEGA) and high-resolution transmission electron microscopy (HR-TEM; JEOL 2000-FX).

Elemental analysis of the Al/CF composites was performed through energy dispersive X-ray spectroscopy (EDS; the EDS detector attached to the SEM microscope) and electron probe microanalyzer (EPMA; CAMECA SX 100).

### 2.4. Thermal and Thermomechanical Characterization

The thermal conductivities of the composite materials were calculated using the following equation [11]:

(2) $$K_{C}=\;\alpha \cdot \rho_{C}\cdot C_{p},$$

where *K_C_* is the TC, *α* is the thermal diffusivity, *ρ_c_* is the density and *C_p_* is the specific heat of the composite measured by a calorimetric measurement. The thermal diffusivity was measured by the flash laser method (NETZSCH LFA 457, MicroFlash) at room temperature. For Al/CF composite materials, due to the anisotropy of the reinforcement, both transverse (parallel to the pressure direction) and in-plane (perpendicular to the pressure direction) thermal diffusivities were measured.

The in-plane CTE was measured using a dilatometry equipment (NETZSCH DIL 402, PC), under argon gas flow. Two thermal cycles were performed between room temperature and 250 °C with 2 °C/min of heating/cooling rate.

### 2.5. Theoretical Models

#### 2.5.1. Thermal Conductivity Model

Theoretical thermal conductivities values of Al/CF and Al/DP composites were calculated using the Hasselman and Johnson model [12] for cylinders Equation (3) and spherical Equation (4) reinforcements, respectively:

(3) $$K_{c}=K_{m}\frac{\left(\frac{K_{r}}{K_{m}}-\frac{K_{r}}{ah_{c}}-1\right)V_{r}+\left(1+\frac{K_{r}}{K_{m}}+\frac{2K_{r}}{ah_{c}}\right)}{\left(1-\frac{K_{r}}{K_{m}}-\frac{K_{r}}{ah_{c}}\right)V_{r}+\left(1+\frac{K_{r}}{K_{m}}+\frac{2K_{r}}{ah_{c}}\right)},$$

(4) $$K_{c}=K_{m}\frac{2\left(\frac{K_{r}}{K_{m}}-\frac{K_{r}}{ah_{c}}-1\right)V_{r}+\left(2+\frac{K_{r}}{K_{m}}+\frac{2K_{r}}{ah_{c}}\right)}{\left(1-\frac{K_{r}}{K_{m}}-\frac{K_{r}}{ah_{c}}\right)V_{r}+\left(2+\frac{K_{r}}{K_{m}}+\frac{2K_{r}}{ah_{c}}\right)},$$

where *K_c_*, *K_m_*, and *K_r_* are TC of composite, matrix, and reinforcement, respectively; *V_r_* and a are the volume fraction and the radius of the reinforcement, respectively, and *h_c_* is the boundary conductance. The key point of the Hasselman and Johnson model is the dependence of TC on the particulate radius a, and the boundary conductance *h_c_* which is the reciprocal of interfacial thermal resistance.

Heat transportation is due to electrons in metal and to phonons in carbon reinforcement like CF or DP. Due to the extremely low free electron concentration in carbon reinforcements, the heat transportation through interfaces between metal and carbon is dominated by phonons. Therefore, the boundary conductance *h_c_* of the composite material could be calculated using the Acoustic Mismatch Model (AMM) [13,14,15] given by Equation (5):

(5) $$h_{C}=\;\frac{1}{2}\rho_{m}C_{m}\frac{\nu_{m}^{3}}{\nu_{r}^{2}}\frac{\rho_{m}V_{m}\rho_{r}V_{r}}{{\left(\rho_{m}V_{m}+\rho_{r}V_{r}\right)}^{2}},$$

where *ρ*, *C,* and *ν* are the density, specific heat capacity, and phonon velocity of the materials, respectively, and *c*, *m,* and *r* denote, respectively, composite, matrix, and reinforcement. The material parameters used for the calculations are tabulated in Table 1.

#### 2.5.2. Coefficient of Thermal Expansion Models

Concerning the calculation of the in-plane CTE, the Kerner model [13,16], given below, was used for composites with diamond reinforcements:

(6) $$\alpha_{C}=\alpha_{m}V_{m}+\alpha_{d}V_{d}+V_{d}V_{m}\left(\alpha_{d}-\alpha_{m}\right)\left(\frac{K_{d}-K_{m}}{V_{m}K_{m}+V_{d}K_{d}+(3K_{d}K_{m}/4G_{m})}\right),$$

where *α_c_*, *α_m_*, and *α_d_* are the CTE of composite, matrix and DP, respectively; *V_m_* and *V_d_* are the volume fraction of matrix and DP, respectively, *K_m_* and *K_d_* are the bulk modulus of matrix and DP, respectively, and G_m_ is the shear modulus of the matrix.

The material parameters used for the calculations are also tabulated in Table 1.

## 3. Results and Discussion

### 3.1. Al/CF Composite Materials

#### 3.1.1. Relative Density

**Figure 3:**
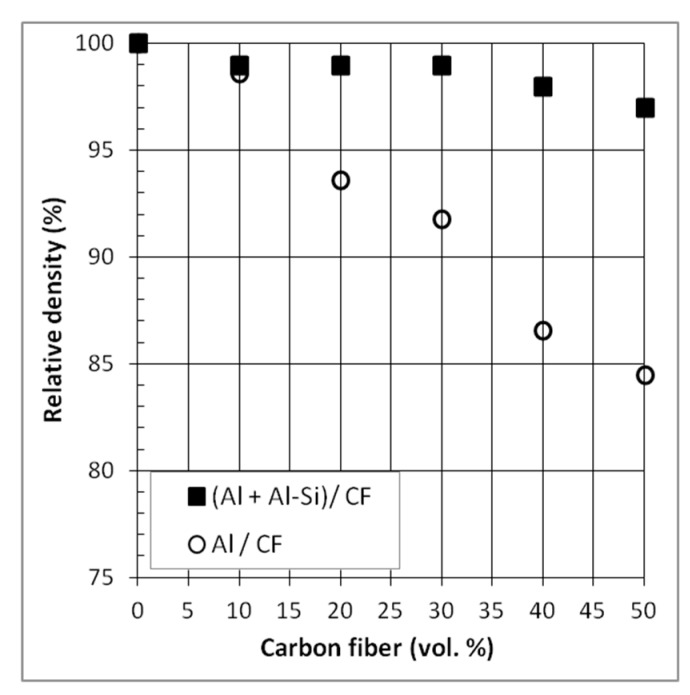
Relative density of Al/CF and (Al + Al-Si)/CF composite materials.

The relative density of the hot-pressed Al/CF composite materials as a function of the reinforcement volume (from 0 to 50 vol.% of CF) is shown in Figure 3. As expected, for the two sets of materials, the relative density decreases when the CF content increases. However, the relative density of set A samples drastically decreases when the volume fraction of CF becomes higher than 20 vol.%. This behavior is related to the increase of porosity located at the intertwining of CFs. However, set B composites, with Al-Si additive, exhibit a higher level of relative density (higher than 97%) than set A. This result confirmed the interest of this semi-liquid process. Indeed, during the sintering with a liquid phase, Al particles as well as CF rearrangement can take place and the Al-Si liquid phase can also flow around the grains and infiltrate the pores created by the intertwining of CF.

#### 3.1.2. Microstructure

**Figure 4:**
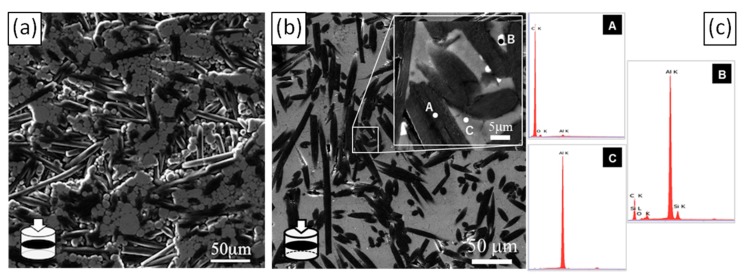
SEM micrographs of (**a**) (Al/CF)_50vol%_ and (**b**) ((Al + Al-Si)/CF)_50vol%_ composites in a plane perpendicular to the hot-pressed direction (in plane) and (**c**) EDS spot scan in ((Al + Al-Si)/CF)_50vol%_ composite.

Figure 4 shows SEM micrographs of sintered composite materials of set A and B with 50 vol.% of CF. It has to be noticed that, whatever the fabrication process (solid or semi-liquid), the CF are randomly oriented in the plane perpendicular to the pressure direction, due to the uniaxial compressive stress imposed by the hot press. The SEM micrographs of set A composite (Figure 4a) show voids at Al-CF and CF-CF interfaces, which is consistent with the decrease of the relative density observed for these types of composite materials (Figure 3). On the contrary, set B composite presents a tight Al-CF interface (Figure 4b). The EDS analysis (Figure 4c) reveals the presence of Si clusters (point B) at the Al-CF interface. These results clearly indicate that, during the sintering process, the presence of liquid Al-Si actually contributes to densify Al/CF composites.

**Figure 5:**
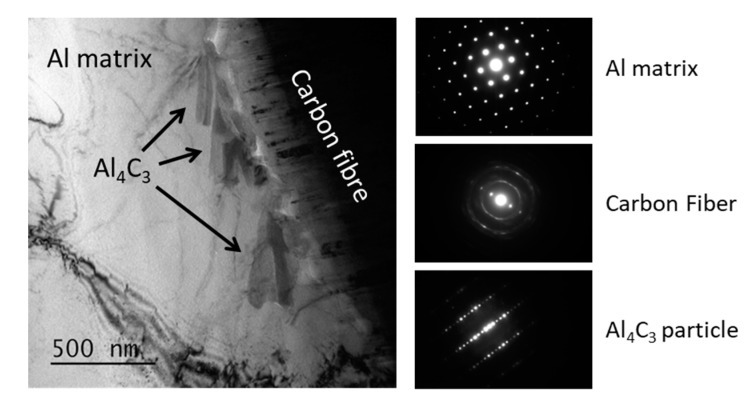
TEM micrographs of (Al + Al-Si)/CF50vol% composite and associated diffraction patterns.

Transmission electron microscopy was performed on (Al + Al-Si)/CF composite reinforced with 50 vol.% of CF (Figure 5). TEM micrograph clearly shows (i) the presence of needle like carbides at the Al-CF interface (it has to be mentioned that these carbides are not present at the Al/CF composite material sintered without a Al-Si liquid phase), and (ii) the absence of porosity at the Al-CF interface.

**Figure 6:**
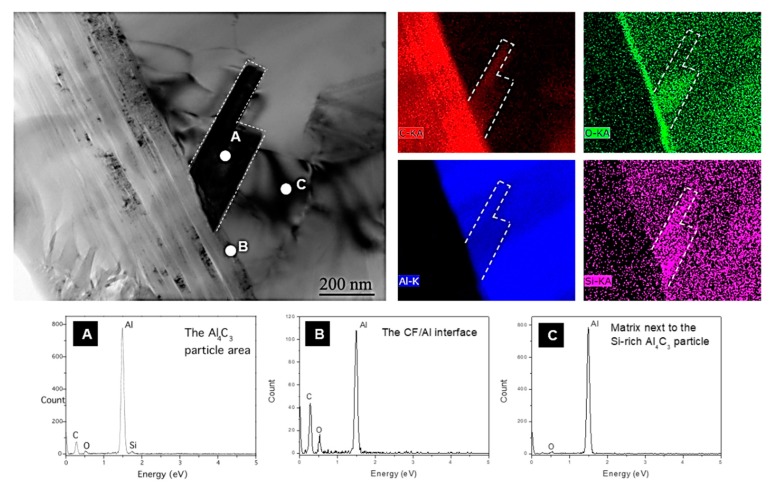
TEM micrograph, EDX mappings and spot analyses of an Al_4_C_3_ carbide crystal in ((Al + Al-Si)/CF)_50 vol.%_ composite. (**A**), (**B**) and (**C**) correspond to the spectrums of the EDX spot analyses in zone A, B and C respectively.

The composition of the needle like carbide has been analyzed in detail. Figure 6 shows a TEM micrograph at the Al-CF interface that contains Al carbide needles and an EDS map and spot analysis of this area. Several points can be outlined from this analysis:

1. The presence of oxygen at the Al-CF interface (Figure 6—point B of the EDS spot) which is consistent with the alumina layer present on the surface of the Al powder particles before sintering.
2. The presence of some dislocations inside the Al matrix and close to the Al-CF interface which can be attributed to the thermo-mechanical stresses induced by the difference of CTE between the CF and the Al matrix.
3. A homogeneous distribution of the Si inside the Al matrix.
4. A higher content of Si and O on the Al_4_C_3_ carbide particles (EDS map and EDS point A). This result is consistent with previous works using a squeeze-casting method to densify Al/Diamond composites material. It seems that the Al-Si eutectic phase segregates to the carbon reinforcement surface and participates in the Al/C bonding [10,20].

#### 3.1.3. Thermal Conductivity

**Figure 7:**
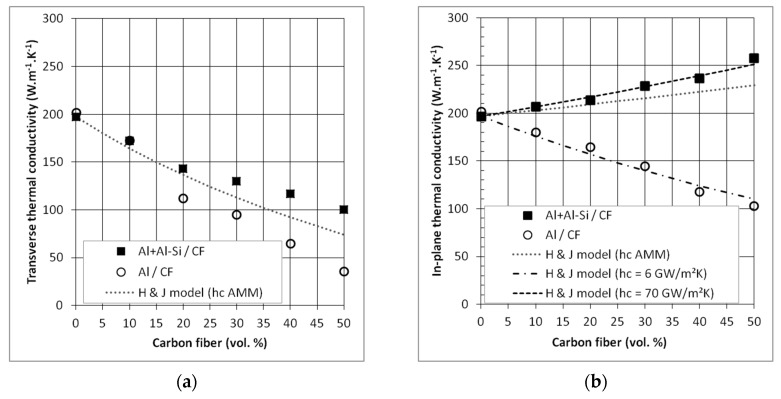
Thermal conductivities of Al/CF and (Al + Al-Si)/CF composites in (**a**) transverse and (**b**) in-plane direction.

Figure 7 shows the TC of Al/CF and (Al + Al-Si)/CF composite materials in transverse and in-plane directions. Indeed, due to the random orientation of the CF in a (*x*, *y*) plane, perpendicular to the pressure direction (z), the Al/CF materials have anisotropic properties.

In the transverse direction (z) (Figure 7a), we measure a decrease of the TC with the increase of CF ratio for both sets of samples. In addition, the TC values of Al/CF composites are lower than the TCs of the (Al + Al-SI)/CF one. Therefore, the behavior of the transverse TC of the composite is related to the intrinsic properties of the reinforcement (see Table 1). The lower thermal conductivity of the Al/CF composites, with respect to the (Al + Al-Si)/CF one, can be attributed to the presence of porosity located at the Al-CF interfaces which increase the thermal boundary resistance.

In the in-plane direction (x, y) (Figure 7b), the TC of the (Al + Al-Si)/CF composite materials increases with the CF ratio and reaches 258 W·m^−1^·K^−1^ for a CF volume fraction close to 50%. It has to be mentioned that, due to the fact that the CF are randomly oriented in this (*x*, *y*) plane, the TC value is equivalent whatever the direction of measurement inside this plane. This TC is slightly higher than that of pure Al (200 W·m^−1^·K^−1^) while the in-plane TC of Al/CF composites decreased with the CF volume fraction. These results confirmed the higher density of composites with Al-Si addition and the creation of an efficient interface that allows an efficient transfer of properties between the matrix and the reinforcement, and then an increase of the thermal conductivity of the composite due to the high TC of the CF in that direction.

We have compared our experimental results to the TC calculated using a Hasselman and Jonshon model (H & J model). In this model, the interfacial thermal resistance between the matrix and the reinforcement was also considered, in order to better describe the TC, through the boundary conductance *h_c_*, which is the inverse of the interfacial thermal resistance.

We used the acoustic mismatch model Equation (5) to evaluate the boundary conductance, *h_c_*. We found a value of *h_c_* equal to 4.8 × 10^7^ W·m^−2^·K^−1^, which is consistent with other values found in the literature [20,21]. This value of interfacial thermal conductance (*h_c_* AMM) does not allow for perfectly fitting the experimental TC of the Al/CF and (Al + Al-Si)/CF composites, as it can be seen in Figure 7. Indeed, the AMM model considers a straight interface with a perfect mechanical contact. For Al/CF composites, the density measurements and the microstructural analysis have shown that some porosities were present at the Al-CF interfaces, which interrupt the thermal flow. In fact, it is possible to fit the experimental curve using an interfacial thermal conductance of 6.0 × 10^6^ W·m^−2^·K^−1^, which is eight times lower than AMM model (corresponding to a higher thermal resistance). For (Al + Al-Si)/CF composites, the measured thermal conductivities are higher than that predicted by the AMM model (Figure 7b). It means that the “quality” of the interface, in terms of transfer of thermal flux, between Al and CF can be considered as very good. For these first calculations with the AMM model, we used only the parameters for Al and CF. However, TEM analyses (Figure 6) have shown the presence of Aluminum carbide (Al_4_C_3_) particles at the Al-CF interfaces. Therefore, the overall interfacial thermal conductance encountered in (Al + Al-Si)/CF composites should take into account an Al-Al_4_C_3_-CF interface (h_Al-Al4C3-CF_). It can be calculated according to Equation (7), using the addition of the thermal resistances of the different interfaces:

(7) $$R_{Al-Al_{4}C_{3}-CF}=\frac{1}{h_{Al-Al_{4}C_{3}-CF}}=\frac{1}{h_{Al-Al_{4}C_{3}}}+\frac{1}{h_{Al_{4}C_{3}-CF}}.$$

The thermal conductance of Al-Al_4_C_3_ (h_Al-Al4C3_) and Al_4_C_3_/CF (h_Al4C3-CF_) has been calculated as equal to 18.6 × 10^7^ W·m^−2^·K^−1^ and 58.2 × 10^7^ W·m^−2^·K^−1^, respectively. Thus, the interfacial thermal conductance of Al-Al_4_C_3_-CF is equal to 14.1 × 10^7^ W·m^−2^·K^−1^. Thereby, the carbide formation at the Al/CF interface would increase the interfacial thermal conductance, previously calculated as 4.8 × 10^7^ W·m^−2^·K^−1^. Using H & J model, the experimental curve can be fitted using *h_c_* = 7.0 × 10^7^ W·m^−2^·K^−1^, a value lower than the Al-Al_4_C_3_-CF conductance. This is in agreement with TEM observations that show that the aluminum carbides do not perfectly cover CF. Therefore, the Al-CF interfacial properties would be a combination of Al-CF and Al-Al_4_C_3_-CF interfacial properties.

In the transverse direction, the interfacial thermal conductance value has no significant influence on the thermal conductivities. Indeed, intrinsic thermal conductivity of CF is significantly lower (10 W·m^−1^·K^−1^) than that of the Al matrix. Therefore, in this direction, the CF reinforcements tend to lower the thermal flow. Consequently, the thermal transfer is mainly ensured by the Al matrix, and the interfacial thermal conductance has less effect on the transverse thermal conductivity of the composite.

#### 3.1.4. Coefficient of Thermal Expansion

**Figure 8:**
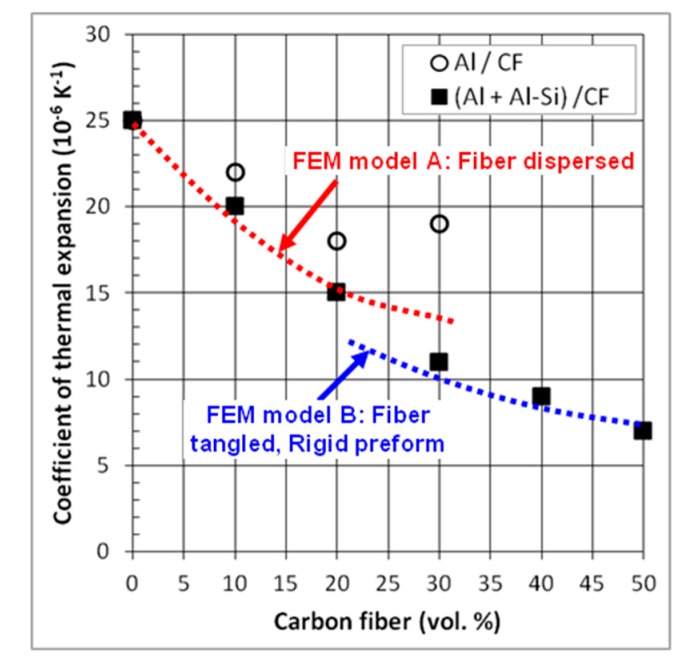
CTE of Al/CF and (Al + Al-Si)/CF composites in-plane direction.

Figure 8 shows the evolution of the CTE as a function of the fiber volume fraction for Al/CF and (Al+Al-Si)/CF composite materials, measured in the (*x*, *y*) plane. The CTE of the Al matrix (25.0 × 10^−6^ K^−1^) is higher than that of pure Al (23.0 × 10^−6^ K^−1^). The CTE of (Al + Al-Si)/CF and Al/CF composite materials decreases more or less linearly with the addition of CF, up to 20 vol.%. Then, for Al/CF composites, the CTE tends to increase again for a CF volume fraction of 30%, and it was no longer measurable for 40 vol.%, due to the deterioration of the composite, associated with its low relative density. For (Al + Al-Si)/CF composites, their CTE continues to decrease as the CF content increases. It reaches 7.0 × 10^−6^ K^−1^ for 50 vol.%. Such a value appears quite interesting given that the heat sink materials must have a coefficient of thermal expansion close to that of the surrounding layers, in order to limit thermal stresses in the package [3,22]. This corresponds to values generally comprised between 2.6 and 7.0 × 10^−6^ K^−1^. Therefore, our (Al + Al-Si)/CF composite with 50 vol.% of CF presents a coefficient of thermal expansion (7.0 × 10^−6^ K^−1^), which could therefore allow it to be used as a heat dissipation material in electronic packages.

The calculation of the CTE values in composite materials is not straightforward. It is known that the CTE does not follow the classical rule of mixtures due to presence of thermal stresses at the matrix–reinforcement interfaces [6,7]. Several models are reported in the literature for the CTE of composite materials containing continuous fibers as reinforcements [23,24]. However, these models cannot be applied for short fiber containing composites. Therefore, we have developed a finite element method (FEM) for calculating the CTE of such composite materials [25]. This FEM is in fact made of two models in order to take into account the percolation threshold of the CF in the Al matrix. Model A corresponds to a composite without CF tangles and model B a composite with tangles of CF. The comparison between the experimental and calculated CTE of (Al + Al–Si)/CF composites is shown in Figure 8. It shows that the separation into two models for calculating the CTE is actually pertinent to correctly take into account the evolution of the CTE with the fiber content. Calculated values of CTE obtained with FEM model B are smaller than those determined by FEM model A. It clearly shows that the tangles of the carbon fiber contribute to further improve the CTE in (Al + Al–Si)/CF composites. In addition, it seems that the remarkable low CTE of the carbon fibers has been transferred into the Al matrix and contributes effectively to the low CTE of the composite materials.

### 3.2. Aluminum/Diamond Particle Composite Materials

#### 3.2.1. Relative Density

**Figure 9:**
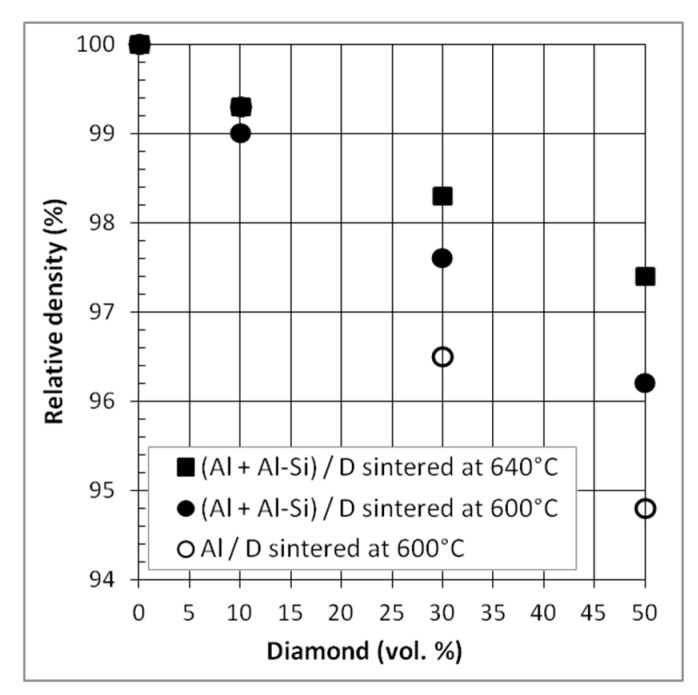
Relative density of Al/DP and (Al + Al-Si)/DP composite materials sintered at 600 °C and/or 640 °C.

The relative density of the composites containing 10, 30, and 50 vol.% of diamond particles is shown in Figure 9. Like for Al/CF composites, the relative density decreases with the increase in reinforcement content but to a much lesser extent (the relative density of the 50 vol.% Al/DP composite is close to 94% compared to 85% for Al/CF). The Al-Si addition in the Al/DP composites not only results in higher relative densities than Al/DP, but also in high relative densities, even for high DP content. The effect of the sintering temperature is also observed for these (Al + Al-Si)/DP composites, with a clear improvement of the sintered densities with the increase of the sintering temperature from 600 °C to 640 °C. A final relative density higher than 97% is obtained for the 50 vol.% (Al + Al-Si)/DP composite sintered at 640 °C. This result confirms the interest of the liquid phase sintering approach.

#### 3.2.2. Microstructure

**Figure 10:**
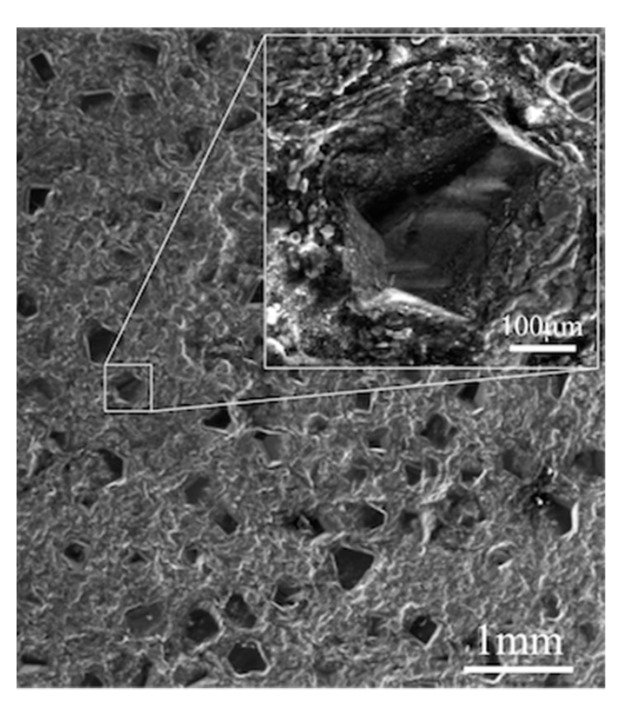
SEM micrograph of (Al + Al-Si)/D50vol% composite sintered at 640 °C.

Figure 10 shows the SEM micrograph of hot-pressed (Al + Al-Si)/DP composites with 50 vol.% of diamond sintered at 640 °C. Homogeneous distributions of diamond in the Al matrix as well as the absence of porosity at the interfaces were observed.

#### 3.2.3. Thermal Conductivity (TC)

**Figure 11:**
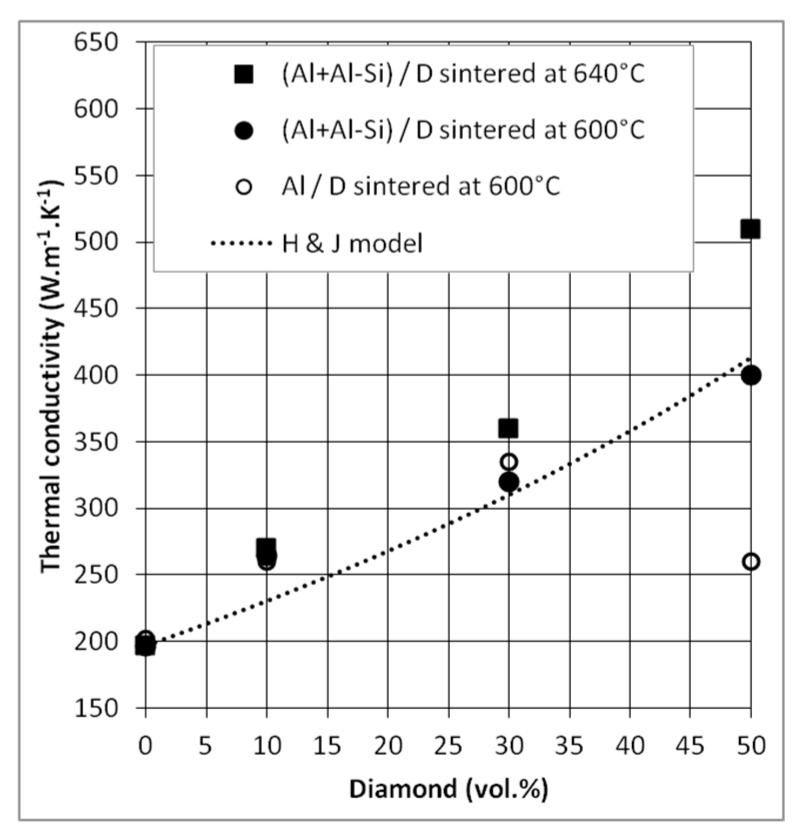
TC of Al/DP and (Al + Al-Si)/DP composites sintered at 600 °C or 640 °C and comparison to Hasselman and Johnson model.

Figure 11 shows the thermal conductivities of Al/DP and (Al + Al-Si)/DP composites sintered at 600 °C and 640 °C. For all composites, TC increases with DP volume fraction, excepted for the Al/DP composite material with 50 vol.% of DP. For this sample, a low TC was obtained, close to 260 W·m^−1^·K^−1^, that can be attributed to the lowest relative density measured for this composite and therefore to the presence of porosities at the Al-DP interface. Conversely, a TC close to 510 W·m^−1^·K^−1^ was measured for the liquid phase sintered composite ((Al + Al-Si)/DP) containing 50 vol.% of DP, which is 2.6 times higher than pure Al (k_Al_ = 197 W·m^−1^·K^−1^). This result is consistent with the thermal conductivity of diamond particles, which is estimated in this study to be close to 1200 W·m^−1^·K^−1^. We also observed a lower increase of the thermal conductivities of (Al + Al-Si)/D composites sintered at 600 °C compared to those sintered at 640 °C, which is, again, consistent with the higher level of densification obtained at that sintering temperature (640 °C).

Experimental TC were compared to the theoretical values, predicted by the Hasselman & Johnson model for spherical reinforcements Equation (4). The thermal conductance used in this model was calculated using the AMM model, like for CF reinforcements Equation (5). A value of *h_c_* = 4.67 × 10^7^ W·m^−2^·K^−1^ was obtained for Al/DP composite materials; this value is consistent with other values reported in the literature [19,20]. However, with this h_c_ value, the model does not allow to fit perfectly our experimental results. Three points can be advanced to explain this difference in behavior: (i) the exact value of the thermal conductivity of these diamond particles is not really known, (ii) the shape of the diamond particles, although isotropic, is not spherical, and (iii) the model is initially designed for small reinforcement volume fractions. As a matter of fact, the deviation of the model predictions increases with the DP volume fraction (the dilute medium assumption is affected when the diamond volume fraction increases). Despite this uncertainty, the evolution of experimental TC of (Al + Al-Si)/DP composites follows that of the model.

#### 3.2.4. Coefficient of Thermal Expansion (CTE)

**Figure 12:**
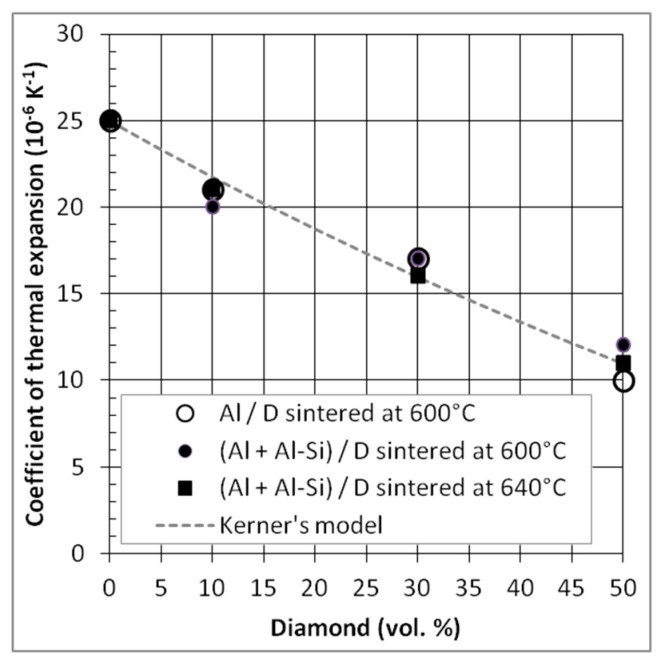
CTE of Al/DP and (Al + Al-Si)/DP composites sintered at 600 °C and/or 640 °C and comparison to CTE values calculated using the Kerner model.

Theoretical and experimental CTE results obtained from Al/DP and (Al + Al-Si)/DP composites, hot-pressed at 600 °C and 640 °C, are shown in Figure 12. The measured CTE of both composite materials decreases linearly with the increase of DP volume fractions from 0% to 50%. Unlike TC, CTE is not affected by the presence of porosity inside the composite materials. CTE of Al/DP is close to the CTE of (Al + Al-Si)/DP. The obtained experimental values are in agreement with Kerner’s model predictions; CTE values calculated with this model are close, up to 50 vol% of DP, to the measured CTE values. It means that the transfer of properties between matrix and reinforcements is actually effective, due to the presence of chemical bondings at these interfaces.

The best results in terms of CTE are obtained for 50 vol.% of diamond reinforcements. The value for the Al/DP composite material is close to 10 × 10^−6^ K^−1^ which represent a strong reduction (40%) relative to pure Al material (α_Al_ = 25 × 10^−6^ K^−1^). Nevertheless, this value is slightly higher than the target value of 7 × 10^−6^·K^−1^ for electronic packaging.

## 4. Conclusions

In the present study, the semi-liquid method has been investigated in order to fabricate Al/CF and Al/DP composite materials. This process involved the presence of a small fraction of liquid phase (Al-Si) during the sintering process. Density, microstructural, thermal and thermo-mechanical properties have been investigated, and it has been shown that:

- Liquid Al-Si phase contributed to decreasing the porosity ratio and optimizing the Al-C interfacial zone for both Al/CF and Al/DP composite materials.
- Si cluster and Al needle like carbide have been analyzed, by TEM, at the Al-CF interface for the Al/CF composite materials.
- Thermal conductivity and coefficient of thermal expansion appear to be strongly related to the composite density and the Al-C interfacial properties.
- Values of thermal conductivity of 258 W·m^−1^·K^−1^ and coefficient of thermal expansion of 7.0 × 10^−6^ K^−1^ were obtained for (Al + Al-Si)/CF composite with 50 vol.% of CF. These values satisfy the typical requirements for heat sink materials.
- Significant increase of the thermal conductivity (510 W·m^−1^·K^−1^) is obtained in the case of (Al + Al-Si)/DP composite materials with 50 vol.% of diamond particles. This thermal conductivity is coupled with a CTE of 10 × 10^−6^ K^−1^.

## Figures and Tables

**Table 1:** Materials parameters used for calculation in the theoretical models [17,18,19,20].

Materials		Al	CF	DP
Density (kg·m^−3^)		2713	2260	3500
Specific heat (J·Kg^−1^·K^−1^)		916	836	512
Size (µm)	Length	50	200	65
	Diameter		10	
Thermal conductivity (W·m^−1^·K^−1^)	Along axis	197	600	1200
	Perpendicular to the axis		10	
CTE (10^−6^ K^−1^)	Along axis	23	−1	1
	Perpendicular to the axis		10	
Poisson ratio		0.34	-	-
Phonon velocity (m·s^−1^)		3620	14,660	13,430
Elastic modulus (GPa)		71	760	1142
Shear modulus (GPa)		26.6	-	535
Bulk modulus (GPa)		68.55	-	442
